# *MIPEP* recessive variants cause a syndrome of left ventricular non-compaction, hypotonia, and infantile death

**DOI:** 10.1186/s13073-016-0360-6

**Published:** 2016-11-01

**Authors:** Mohammad K. Eldomery, Zeynep C. Akdemir, F.-Nora Vögtle, Wu-Lin Charng, Patrycja Mulica, Jill A. Rosenfeld, Tomasz Gambin, Shen Gu, Lindsay C. Burrage, Aisha Al Shamsi, Samantha Penney, Shalini N. Jhangiani, Holly H. Zimmerman, Donna M. Muzny, Xia Wang, Jia Tang, Ravi Medikonda, Prasanna V. Ramachandran, Lee-Jun Wong, Eric Boerwinkle, Richard A. Gibbs, Christine M. Eng, Seema R. Lalani, Jozef Hertecant, Richard J. Rodenburg, Omar A. Abdul-Rahman, Yaping Yang, Fan Xia, Meng C. Wang, James R. Lupski, Chris Meisinger, V. Reid Sutton

**Affiliations:** 1 Department of Molecular and Human Genetics, Baylor College of Medicine, Houston, TX 77030 USA; 2 Institute of Biochemistry and Molecular Biology, ZBMZ and BIOSS Centre for Biological Signalling Studies and Faculty of Medicine, University of Freiburg, 79104 Freiburg, Germany; 3 Texas Children’s Hospital, Houston, TX 77030 USA; 4 Department of Pediatrics, Tawam Hospital, Al Ain, 15258 United Arab Emirates; 5 Human Genome Sequencing Center, Baylor College of Medicine, Houston, TX 77030 USA; 6 Department of Pediatrics, University of Mississippi Medical Center, 2500N State St, Jackson, MS 39216 USA; 7 Baylor Miraca Genetics Laboratories, Baylor College of Medicine, Houston, TX 77030 USA; 8 Medical Genetics Center, Jiang Men Maternity and Childhealth Care Hospital, Jiang Men, 529000 China; 9 Huffington Center on Aging, Baylor College of Medicine, Houston, TX 77030 USA; 10 Human Genetics Center, University of Texas Health Science Center at Houston, Houston, TX 77030 USA; 11 Radboud Center for Mitochondrial Medicine, Department of Pediatrics, RadboudUMC, 6500HB Nijmegen, Netherlands; 12 Department of Pediatrics, Baylor College of Medicine, Houston, TX 77030 USA

## Abstract

**Background:**

Mitochondrial presequence proteases perform fundamental functions as they process about 70 % of all mitochondrial preproteins that are encoded in the nucleus and imported posttranslationally. The mitochondrial intermediate presequence protease MIP/Oct1, which carries out precursor processing, has not yet been established to have a role in human disease.

**Methods:**

Whole exome sequencing was performed on four unrelated probands with left ventricular non-compaction (LVNC), developmental delay (DD), seizures, and severe hypotonia. Proposed pathogenic variants were confirmed by Sanger sequencing or array comparative genomic hybridization. Functional analysis of the identified MIP variants was performed using the model organism *Saccharomyces cerevisiae* as the protein and its functions are highly conserved from yeast to human.

**Results:**

Biallelic single nucleotide variants (SNVs) or copy number variants (CNVs) in *MIPEP*, which encodes MIP, were present in all four probands, three of whom had infantile/childhood death. Two patients had compound heterozygous SNVs (p.L582R/p.L71Q and p.E602*/p.L306F) and one patient from a consanguineous family had a homozygous SNV (p.K343E). The fourth patient, identified through the GeneMatcher tool, a part of the Matchmaker Exchange Project, was found to have inherited a paternal SNV (p.H512D) and a maternal CNV (1.4-Mb deletion of 13q12.12) that includes *MIPEP*. All amino acids affected in the patients’ missense variants are highly conserved from yeast to human and therefore *S. cerevisiae* was employed for functional analysis (for p.L71Q, p.L306F, and p.K343E). The mutations p.L339F (human p.L306F) and p.K376E (human p.K343E) resulted in a severe decrease of Oct1 protease activity and accumulation of non-processed Oct1 substrates and consequently impaired viability under respiratory growth conditions. The p.L83Q (human p.L71Q) failed to localize to the mitochondria.

**Conclusions:**

Our findings reveal for the first time the role of the mitochondrial intermediate peptidase in human disease. Loss of MIP function results in a syndrome which consists of LVNC, DD, seizures, hypotonia, and cataracts. Our approach highlights the power of data exchange and the importance of an interrelationship between clinical and research efforts for disease gene discovery.

**Electronic supplementary material:**

The online version of this article (doi:10.1186/s13073-016-0360-6) contains supplementary material, which is available to authorized users.

## Background

Left ventricular non-compaction (LVNC) is a heterogeneous disorder that may present with heart failure, arrhythmia, and systemic embolism [1]. Failure to develop compact myocardium in the early embryo results in LVNC with the underlying genetic basis identified in around 30–50 % of individuals with LVNC [1]. Different modes of inheritance have been described for LVNC, including autosomal dominant, X-linked, and mitochondrial inheritance. Among them, the most prevalent form is the autosomal dominant inheritance pattern (70 % of all cases where the genetic basis is known) with incomplete penetrance [1, 2].

Mechanisms that have been implicated in LVNC include variants in genes encoding the substructures of the sarcomere in cardiomyocytes. For example, variants in *MYH7* (MIM 613426) and *MYBPC3* (MIM 615396) both lead to a disruption of myosin function [3, 4]. Additionally, variants in *ACTC1* (MIM 613424), *TNNT2* (MIM 601494), *TPM1* (MIM 611878) and *DTNA* (MIM 604169) cause dysfunction of actin, troponin, tropomyosin, and dystrobrevin, respectively [4–7]. Moreover, variants in *LDB3* (MIM 601493), a gene that plays a role in the maintenance of structural integrity of cardiomyocytes, has been associated with LVNC [8]. Recently, variants in *MIB1* (MIM 615092) have been identified to cause abnormal cardiac trabeculations through dysregulation of the NOTCH pathway [9]. Furthermore, truncations and missense variants of *TAZ* (MIM 302060), a mitochondrial transacylase, have been associated with X-linked dominant LVNC in male patients with Barth syndrome [7, 10]. In many cases of LVNC, however, the underlying genetic bases remain unknown.

We have identified several single nucleotide variants (SNVs) in *MIPEP* in patients with LVNC. Using whole exome sequencing (WES), a homozygous missense SNV in *MIPEP* was identified in a child from a consanguineous family, two compound heterozygous SNVs were found in individuals from two unrelated, non-consanguineous families, and a paternally inherited SNV and maternally inherited 1.4-Mb deletion copy number variant (CNV) was found in a fourth child. Although the aforementioned searches were based upon finding damaging variants in the same gene, remarkably the predominant clinical features shared by all four subjects included LVNC, developmental delay (DD), seizures, and hypotonia; three experienced infantile/early childhood death secondary to cardiomyopathy.

**Fig. 1:**
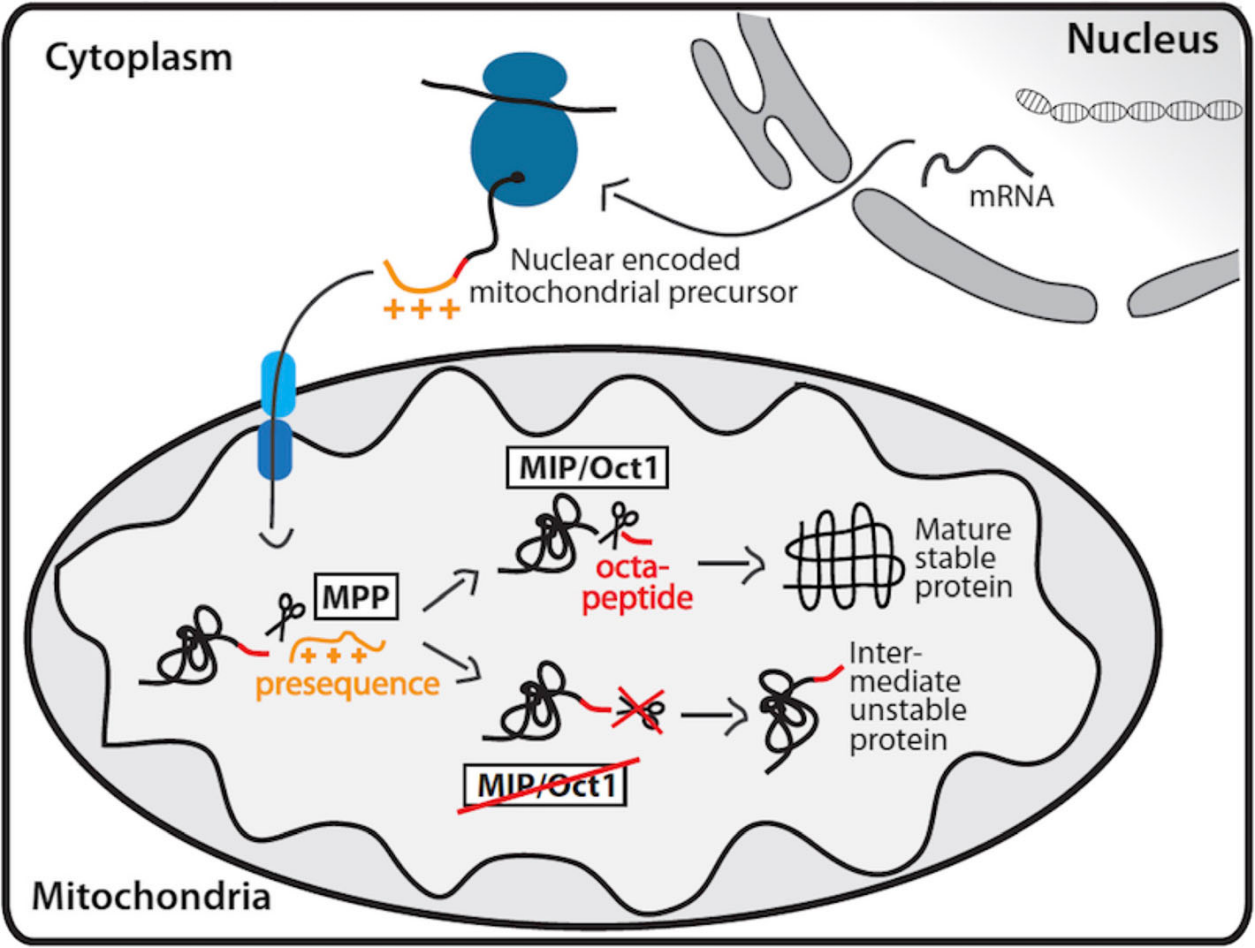
Import of nuclear-encoded proteins into mitochondria and their processing. This process is guided by N-terminal presequences that direct import across the mitochondrial outer and inner membranes through translocons. Precursor processing by MPP and MIP/Oct1 removes the presequence and an additional octapeptide resulting in the mature, stable protein

*MIPEP* encodes the mitochondrial intermediate peptidase (MIP in human, Oct1 in yeast) [11–13]. A vast majority of mitochondrial proteins are encoded by the nuclear DNA. These mitochondrial preproteins are then translated on cytosolic ribosomes and imported post-translationally. Approximately 70 % of these preproteins use N-terminal targeting signals (presequences) for import and translocation across the mitochondrial membranes [14]. Upon entry into the mitochondrial matrix these presequences are cleaved by specialized proteases, the mitochondrial presequence proteases. The major part of the presequence is cleaved by the mitochondrial processing peptidase (MPP). However, approximately one quarter of preproteins require a secondary processing that is carried out by the mitochondrial intermediate peptidase MIP/Oct1 or mitochondrial X-prolyl aminopeptidase 3 (XPNPEP3), also known as intermediate cleaving peptidase Icp55 in yeast and plants [13–16]. MIP/Oct1 removes an additional octapeptide after MPP cleavage (Fig. 1), while XPNPEP3/Icp55 removes a single amino acid [16]. All proteases involved in presequence cleavage are highly conserved from yeast to human, evidence of their fundamental role in mitochondrial biogenesis.

*MIPEP* (MIM 602241, NM_005932) [17, 18] is comprised of 19 exons and maps to chromosome 13q12.12 [19]. MIP is expressed at high levels in heart, brain, skeletal muscle, and pancreas, all tissues that require a significant amount of energy and therefore are dependent on efficient mitochondrial ATP production [19–21]. Furthermore, these tissues are often affected in individuals with various mitochondrial disorders, including MELAS (MIM 540000) and Pearson marrow-pancreas syndrome (MIM 557000) [22, 23]. A broad range of cardiomyopathy disorders have been linked to mitochondrial dysfunction [21, 24, 25]. Here, we report a clinical syndrome of LVNC, DD, seizures, hypotonia, cataracts, and infantile death associated with *MIPEP* dysfunction.

## Methods

### Sequencing

The first three patients initially had clinical WES performed in the Whole Genome Laboratory (WGL), a part of Baylor Genetics Laboratories at Baylor College of Medicine [26]. The coding exons of approximately 20,000 genes were targeted by WES [26, 27] with 130× average depth-of-coverage and >95 % of the targeted bases with >20 reads. The post-processing of raw sequence data was performed using the Mercury pipeline [28]. First, the raw sequencing data (bcl files) were converted to fastq files using Casava. Next, mapping of short reads to the human genome reference sequence (GRCh37) was performed by the Burrows-Wheeler Alignment (BWA) tool. Recalibration and variant calling were then performed using GATK [29] and the Atlas2 suite, respectively [30]. The Mercury pipeline is available in the cloud via DNANexus (http://blog.dnanexus.com/2013-10-22-run-mercury-variant-calling-pipeline/). Any individuals or families in whom clinical WES did not identify a molecular diagnosis in known human disease genes were contacted for possible enrollment in the Baylor-Hopkins Center for Mendelian Genomics (BHCMG) Project for further research analysis of WES data and/or WES of additional family members. The first patient enrolled in the BHCMG Project had *MIPEP* prioritized as a potential candidate gene based on the clinical impression of a mitochondrial disorder. Upon further communication with the clinical exome laboratory, two additional individuals with biallelic variants in *MIPEP* were identified and found clinically to have been referred with a diagnosis of LVNC cardiomyopathy (although the phenotype was not a criteria for the search). The study was approved by the institutional review board (IRB) of Baylor College of Medicine. Additionally, we Sanger sequenced *MIPEP* in 11 individuals aged younger than 5 years with cardiomyopathy and unknown molecular diagnoses at the Baylor Genetics Laboratory.

### Experimental methods

#### Yeast strains and growth conditions

The *Saccharomyces cerevisiae* strains used in this study are derived from BY4741 *oct1Δ* (*Mat*a; *his3Δ1*; *leu2Δ0*; *met15Δ0*; *ura3Δ0*; YKL134c::kanMX4) and YPH499 (*MAT*a, *ade2-101*, *his3-*∆*200*, *leu2-*∆*1*, *ura3-52*, *trp1-*∆*63*, *lys2-801*). For re-expression of Oct1 in the *oct1Δ* strain, the open reading frame under its endogenous promoter and terminator region was cloned into the pRS413 expression vector [12]. Mutations were introduced using site-directed mutagenesis. All plasmids were sequenced and subjected to in vitro transcription/translation in the presence of [^35^S]methionine and analyzed by SDS-PAGE/autoradiography as quality control. The obtained strains were grown on minimal medium (6.7 % (w/v) yeast nitrogen base without amino acids, 2 % glucose (w/v), 0.77 % Complete Supplement Mixture minus histidine).

#### Mutant generation by *plasmid shuffling*

To enable analysis of mutations in vivo under respiratory conditions, the Oct1 protein was expressed from the pRS416 plasmid (*ura3*). Subsequently, the genomic copy of *OCT1* was deleted by homologous recombination. The strain was then transformed with a plasmid encoding Oct1 (pRS413_Oct1) or Oct1 with point mutations resulting in the following amino acid exchanges: L83Q, L339F, K376E, N575D, L645R, and N666* (Additional file 1: Figure S1). All *OCT1* variants were expressed under the endogenous *OCT1* promoter and terminator regions and carried a C-terminal HA tag. When the cells are grown on 5-fluoroorotic acid (5-FOA), the *ura3* gene product converts the 5-FOA into a toxic compound and the cells are selected for pRS416 plasmid loss (*plasmid shuffling*). Transformation with pRS413_Oct1^N575D^, pRS413_Oct1^L645R^ and pRS413_Oct1^N666*^ did not result in viable yeast cells. Of the obtained yeast strains expressing Oct1 mutants L83Q, L339F, and K376E, five independent clones of each mutant and four independent clones of the wild type (Oct^WT^) were tested for growth defects. All clones analyzed showed the same growth behavior. Two to three clones were selected and mitochondria isolated, all of which showed the accumulation of processing intermediates of Oct1 substrates.

For growth tests yeast cells were grown overnight in 5 ml YPG medium at 24 °C. Cell numbers (OD_600_) were measured and adjusted and tenfold serial dilutions were spotted on YPD and YPG agar plates. Plates were incubated at the indicated temperatures.

#### Isolation of mitochondria

For isolation of mitochondria, cells were grown at 24 °C on fermentable medium (1 % (w/v) yeast extract, 2 % (w/v) bacto peptone, 2 % (w/v) sucrose, pH 5.0) or non-fermentable medium (3 % (w/v) glycerol instead of sucrose). Strains expressing the mutant Oct1 proteins (L339F, K376E) were shifted for 10 h to 37 °C prior to isolation of mitochondria. Cells were harvested in the logarithmic growth phase (OD_600_ 1.0–1.5) and mitochondria isolated by differential centrifugation as described previously [31]. Aliquots were stored in SEM buffer (250 mM sucrose, 1 mM EDTA, 10 mM MOPS-KOH, pH 7.2) at −80 °C. Protein levels were analyzed by SDS-PAGE and immunodecoration according to standard protocols.

#### *In organello* import and processing of Oct1 substrate proteins

Radiolabeled precursor proteins were generated by in vitro transcription/translation in rabbit reticulocyte lysates (Promega) in the presence of [^35^S]methionine and incubated with 50 μg isolated mitochondria for the indicated time periods in import buffer (10 mM MOPS/KOH, pH 7.2, 3 % (w/v) bovine serum albumin, 250 mM sucrose, 5 mM MgCl_2_, 80 mM KCL, 5 mM KP_i_, 2 mM ATP, and 2 mM NADH) [31]. As a control for specific import into mitochondria, membrane potential (Δψ) was dissipated prior to the import reaction by the addition of 1 μM valinomycin, 20 μM oligomycin, and 8 μM antimycin A (supplied as AVO mix (1 % (v/v)). All reactions were stopped by addition of 1 % (v/v) AVO. Samples were then placed on ice and treated with 50 μg/ml proteinase K for 10 min. After addition of 2 mM PMSF (phenylmethylsulfonyl fluoride) mitochondria were washed with SEM buffer and subjected to SDS-PAGE [31]. Imported and processed precursor proteins were monitored by digital autoradiography of vacuum-dried electrophoresis gels. All import experiments were reproduced at least two times and with at least two independent cell clones.

## Results

### Genomic analysis

**Fig. 2:**
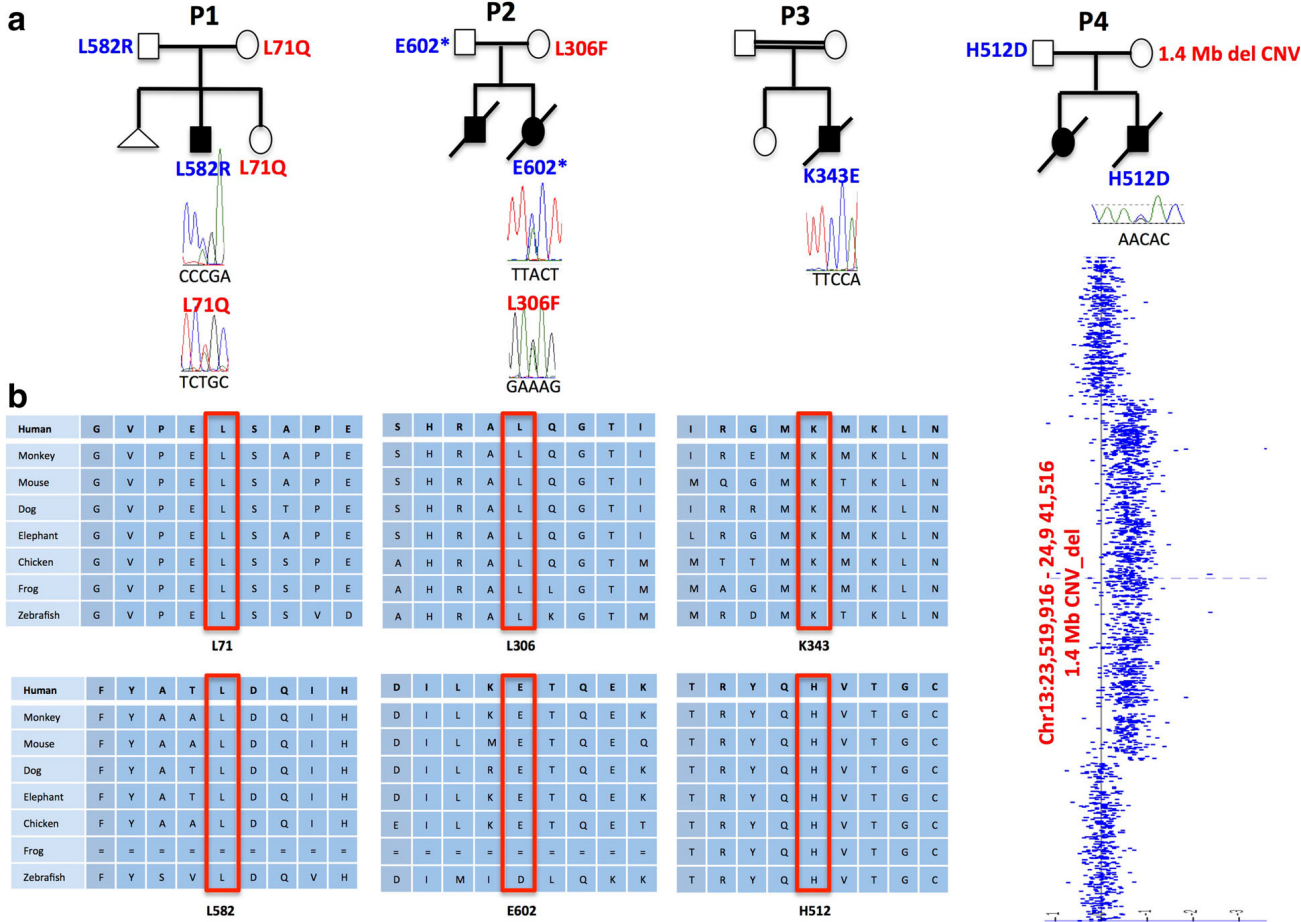
**a** Pedigree structure and segregation analysis for *MIPEP* variants in four families. **b** Evolutionary conservation of *MIPEP* variants among different species at the variant positions found in the study subjects

WES data from patient 1 was transferred to BHCMG and re-analyzed for SNVs in genes not presently associated with human disease in an attempt to determine the molecular underpinnings of the phenotype. Potentially pathogenic compound heterozygous variants (p.L582R/p.L71Q) were identified in *MIPEP*; given what is known about the biology of MIP, this was further pursued as the best potential candidate gene. No other candidate genes with variants bioinformatically predicted to be damaging and conveying a similarly matching phenotype were identified. Upon exchange of our data with the clinical exome laboratory, we identified two more patients with variants in *MIPEP* who both coincidentally had a similar phenotype; patient 2 was found to have compound heterozygous variants (p.E602*/p.L306F) and patient 3 had a homozygous variant (p.K343E). We next submitted *MIPEP* to the GeneMatcher tool, a part of the Matchmaker Exchange project (https://genematcher.org) [32, 33], and successfully matched one other patient with a paternally inherited *MIPEP* SNV (p.H512D) and maternally inherited 1.4-Mb deletion copy number variant (CNV) encompassing the entire gene. Sanger sequencing in the probands and family members confirmed the variants’ presence and their biallelic nature in the probands (Fig. 2). Due to the lack of availability of parental DNA for patient 3, Sanger sequencing of the homozygous variant (p.K343E) was followed by digital droplet PCR to confirm that the variant was indeed homozygous (Additional file 2: Figure S2).

In total, we identified four individuals from four unrelated families with one homozygous and three compound heterozygous variants in *MIPEP* ( 1). Given that patient 1 with LVNC and cardiomyopathy was initially suspected to have a mitochondrial disorder, *MIPEP* was prioritized to be an excellent candidate gene given its fundamental role in mitochondrial biogenesis. *MIPEP* is the strongest candidate among screened variants because the nonsynonymous variants in *MIPEP* are evolutionarily conserved (Fig. 2) and predicted to be deleterious by Mutation Taster, SIFT, PolyPhen2, and CADD (Table 1). Additionally, in our internal Baylor-CMG research database with ~5000 patients, we did not identify any homozygous or compound heterozygous variants in *MIPEP* in subjects who presented with other phenotypes. We also searched our clinical exome laboratory database, which also included ~5000 clinical exomes, and did not find any other individuals with homozygous and compound heterozygous *MIPEP* variants. Upon examination of the 1000 Genome Project, exome variant server (of the Exome Sequencing Project), and Exome Aggregation Consortium (ExAC) databases, we observed that four out of six variants (p.L582R, p.L71Q, p.E602*, and p.K343E) are novel and two variants (p.L306F and p.H512D) are rare, observed in the ExAC database in the heterozygous state at a frequency of 8.2 × 10^−6^ and 3.2 × 10^−5^, respectively. The heterozygous maternal deletion of patient 4 was identified by array comparative genomic hybridization (Affymetrix CytoScan® HD) and encompasses 1.4 Mb at 13q12 (chr13: 23,519,916–24,941,516, hg19) (Fig. 2; Additional file 3: Figure S3).

**Table 1:** *MIPEP* variants in four unrelated patients from four unrelated families

Patient ID	P1	P2	P3	P4
Zygosity	Compound heterozygous	Compound heterozygous	Homozygous	Compound heterozygous
Nucleotide change(s)	c.1745 T > G; c.212 T > A	c.916C > T; c.1804G > T	c.1027A > G	c.1534C > G; NA
Protein change(s)	p.L582R; p.L71Q	p.L306F; p.E602*	p.K343E	p.H512D; NA
db SNP ID(s)	NA	rs143912947, NA	NA	NA
ExAC frequency	NA	8.2 × 10^−6^, NA	NA	3.2 × 10^−5^, NA
Mutation Taster	D	D	D	D, NA
SIFT	D	D	D	D
PolyPhen-2	0.99, 1.00	0.98, NA	0.97	1.00, NA
CADD 1.0 (Phred-like)	29.9, 28.0	29.5, 48	28.5	33, NA
Metabolic myopathy features	Examination of skeletal muscle showed: 1. Moderate variation in fiber size with type 1 fiber predominance 2. Many fibers with increase in subsarcolemmal oxidative activity 3. Increased mitochondria in many fibers by trichrome stain 4. Marked mitochondrial proliferation and pleomorphism on electron microscopy 5. Marked increase in lipid droplets on electron microscopy	Examination of quadriceps muscle by light microscopy showed: 1. Mild variation in fiber size; type 1 fiber predominance with type 1 to type 2 fiber ratio of 70:30 2. Diffuse, moderate to marked increase in glycogen stores on PAS special stain Electron microscopy showed: membrane-bound glycogen deposits; diffuse, mild to moderate increase in lipid droplets on oil-red-O special stain and no increase in oxidative enzyme staining; no increase in mitochondria or mitochondrial pleomorphism, no evidence for a dystrophic process	NA	Light and electron microscopic findings of the skeletal muscle (diaphragm) and cardiac muscle showed: 1. Numerous lipid droplets, glycogen deposition (especially cardiac muscle), and large aggregates of mitochondria. 2. In the skeletal myofibers, the aggregates of mitochondria were often adjacent to vessels. Many of the mitochondria were quite enlarged and had bloated vesicular cristae 3. The ventricles showed thick trabeculae that spanned the lumen and focal clefts in their thick walls. The myofibers swirled and interlaced together. A few, scattered nuclei were enlarged and box-car shaped. Cross striations were well-preserved

CADD (Phred-like) scores ≥20 indicate the variants are among the top 1 % of the most deleterious variants in the genome

*D* damaging, *NA* not applicable, *PAS* periodic acid–Schiff

### Clinical spectrum

Retrospective clinical analyses delineated a consistent phenotype of cardiomyopathy, LVNC, seizures, and hypotonia/developmental delay in all subjects with infantile or early childhood death. Patient 1 was born full term by vaginal delivery after an uncomplicated pregnancy. By 5 months of age, he presented with failure to thrive. At 5.5 months of age, he was diagnosed with LVNC and ECG showed Wolf–Parkinson–White (WPW) syndrome. His family history is notable for a paternal uncle with a history of supra-ventricular tachycardia and maternal great-aunt with early myocardial infarction (29 years of age) but was otherwise unremarkable. His father’s family is of Scottish/Mexican/Native American ancestry and mother’s family is of Colombian/Native American ancestry. There is no known consanguinity. Physical examination revealed length, weight, and head circumference below the fifth centile and he had a wide mouth and bulbous nasal tip, demonstrated tongue-thrusting, and was hypotonic with head lag. Brain MRI showed microcephaly with prominent extra-axial cerebrospinal fluid (CSF) spaces. Evaluations revealed an anion gap metabolic acidosis (anion gap = 25; reference range 3–11 mEq/L) with lactate of 3.2 mmol/L (reference range 0.7–2.1 mmol/L). Plasma amino acid analysis revealed slightly elevated alanine of 553 μmol/L (reference range 103–528 μmol/L) but was otherwise unremarkable. Skeletal muscle biopsy showed evidence for mitochondrial proliferation and lipid droplets by electron microscopy (Table 1). Electron transport chain (ETC) analysis of muscle at a CLIA-certified laboratory showed reductions in several respiratory chain complex activities, although not sufficiently low to satisfy a minor criterion of the modified Walker criteria for the diagnosis of a respiratory chain disorder (Additional file 4: Table S1) [34]. His hypotonia evolved into hypertonia and he has continuous abnormal movements and dystonic posturing. His EEG was normal. He also developed multiple gastrointestinal symptoms, including intermittent vomiting and constipation. He is alive at 4.5 years of age.

Patient 2 was a product of full-term gestation and pregnancy was uncomplicated. A cataract was noted in the left eye shortly after birth and was removed at 3 months of age. She was irritable and fed poorly in the first months of life and had an upper endoscopy at 9 months of age that revealed eosinophilic esophagitis. At 11 months of age she presented with poor feeding and fatigue and was diagnosed with LVNC and dilated cardiomyopathy. Family history was significant for an older brother who had cataracts and infantile spasms and died unexpectedly at 14 months of age of unknown cause. Parents are of European ancestry with no known consanguinity. On physical exam, she had significant hypotonia and global developmental delay. Clinical testing for mitochondrial and other genetic disorders was performed throughout her lifetime and failed to identify a cause for LVNC and hypotonia. She was listed for heart transplant and had a Berlin assist device inserted as a bridge to transplant after worsening cardiac ejection fraction. She subsequently developed uncontrollable seizures. The Berlin device was removed and she died at the age of 2 years. An autopsy revealed diffuse neuronal loss with parenchymal rarefaction and cortical/white matter gliosis involving the frontal cortex, ventral forebrain, and pontine tegmentum (likely secondary to poor brain perfusion from heart disease). LVNC with dilated cardiomyopathy was noted and quadriceps muscle biopsy revealed features of metabolic myopathy (Table 1).

Patient 3 was born at 36 weeks gestation due to pre-term labor. The pregnancy had been uncomplicated. The parents are first-degree cousins from Egypt. There are no similar medical problems in the family. At the age of 2.5 months he had feeding problems with failure to thrive. He was not focusing and exhibited no social smile. At the ages of 5 and 10 months, he was admitted to the hospital for respiratory problems, metabolic acidosis (with lactates of 4.4 and 11.1, respectively; reference range 0.7–2.1 mmol/L) and transient elevation in liver enzymes (alanine transaminase (ALT) 357 U/L, aspartate transaminase (AST) 428 U/L; reference range ALT 6–45 U/L, AST 20–60 U/L). At the age of 9 months, echocardiogram revealed left ventricular hypertrophy without left ventricular outflow tract obstruction and a small secundum atrial septal defect with left to right shunt. The biventricular function was normal. On physical examination, he was noted to have a long philtrum. There was opisthotonus and severe head lag when pulled to sit. By the age of 10 months he developed microcephaly and seizures. Clinical mitochondrial ETC studies from skin biopsy showed mild reductions in all mitochondrial complexes except for complex II and complex V, which were normal (Additional file 4: Table S1). On brain MRI at 10 months, diffusion-weighted images showed a bilateral and symmetrical increase in signal intensity of the basal ganglia, involving mainly the lentiform nucleus. The periventricular white matter showed hyperintense signal in T2-weighted images, interpreted as either normal myelination processes or changes reflecting neurodegenerative processes or a metabolic disorder. The spectroscopy sample on the basal ganglia showed signs of neuronal loss or degradation. He died at 11 months of age.

Patient 4 was delivered by Cesarean section at 33 weeks gestation due to maternal preeclampsia and fetal decelerations. At birth, he was noted to have significant respiratory depression and was intubated. Seizures began within the first hour of life. Physical examination revealed a gallop rhythm and an echocardiogram demonstrated severe biventricular hypertrophic cardiomyopathy. He had dysmorphic features including deep-set eyes, anteverted nares, depressed nasal bridge, midface hypoplasia, severe micrognathia, facial asymmetry, and an accessory palmar crease on the right hand. He was diagnosed with congenital hyperinsulinemia (blood glucose 20 mg/dL; reference range 70–110 mg/dL) and lactic acidosis (8.9–10.4 mmol/L; reference range 0.7–2.1 mmol/L). Laboratory studies showed significant elevations in alanine, glutamine, and proline that were likely to be consistent with liver disease and lactic acidemia. Urine organic acid analysis showed elevated lactate, pyruvate, ketones, and intermediates of the Krebs cycle consistent with lactic acidosis and ketosis. On day 8 of life he was diagnosed with microcolon. By day 14 of life, he required increased ventilatory support; despite this, blood gases continued to worsen with a persistent lactic acidosis and on day 19 of life he expired. Autopsy demonstrated multiple anomalies including rhombencephalosynapsis, narrow bowel, dilated urinary bladder and ureters, small lungs, and massive thick-walled heart. The ventricles showed thick trabeculae that spanned the lumen and thick walls with underlying non-compaction and focal clefts. Additional cardiac findings included patent ductus arteriosus, small membranous ventricular septal defect, congestive heart failure, and pericardial edema. Light and electron microscopic findings of skeletal muscle (diaphragm) and cardiac muscle showed numerous lipid droplets, glycogen deposition (especially cardiac muscle), and large aggregates of mitochondria with bloated vesicular cristae (Table 1). Family history is significant for a previous female infant that presented with cardiomyopathy in the immediate postnatal period and subsequently expired by 16 days of life; it is unknown if she had LVNC or other features such as hypotonia and cataracts.

### Functional analyses

**Fig. 3:**
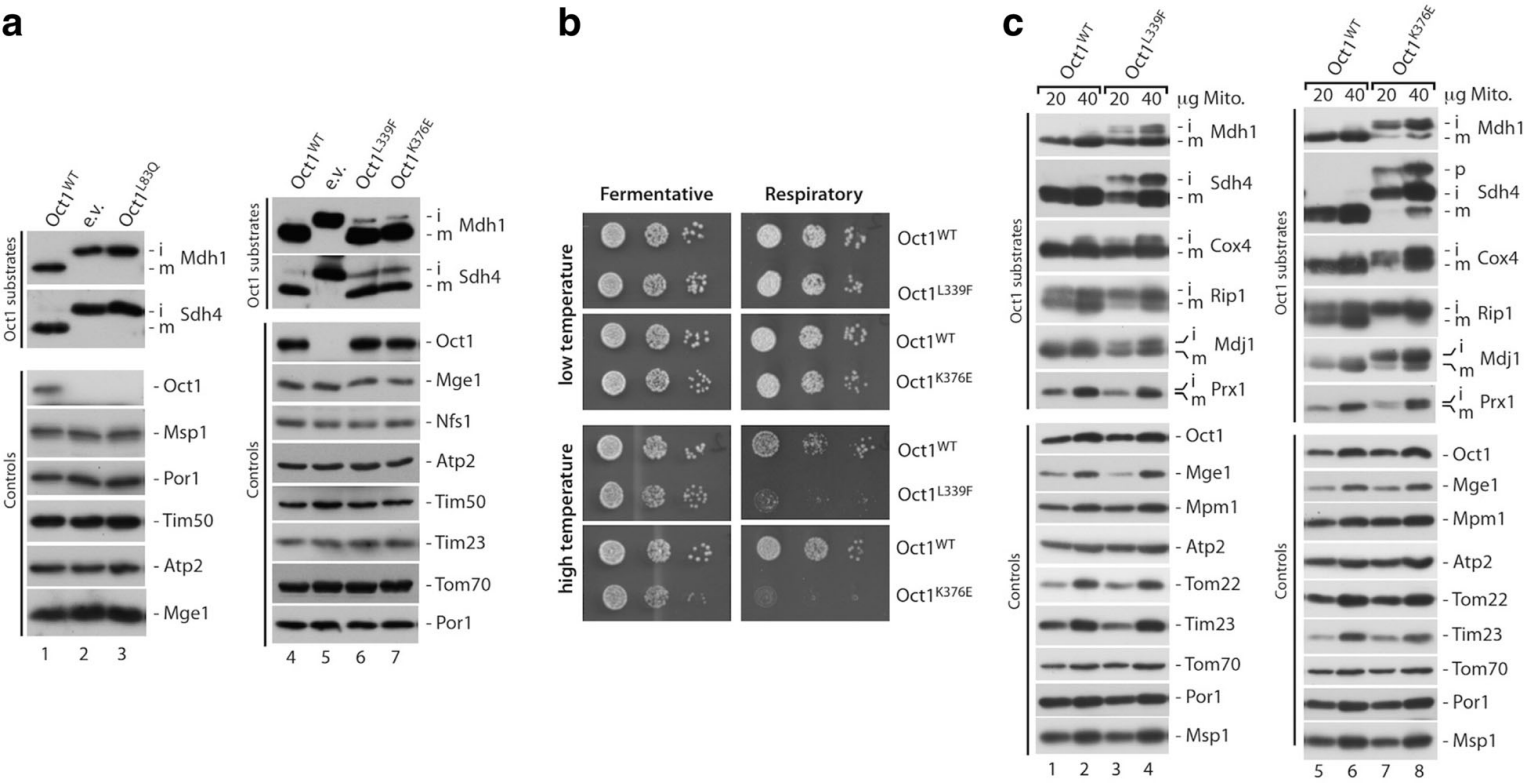
In vivo analysis of *MIPEP*-derived SNVs in the homologous Oct1 protein from *S. cerevisiae*. **a** Immunoblot analysis of mitochondria isolated from *oct1Δ* yeast cells transformed with plasmids encoding wild-type Oct1 (*Oct1* ^*WT*^) or Oct1 mutants (L83Q, L339F, K376E) under the endogenous promoter or the empty control plasmid (*e.v.*). Cells were grown at 24 °C on a fermentable carbon source prior to organelle isolation. **b** Growth of yeast strains expressing Oct1^WT^ or mutants Oct1^L339F^ and Oct1^K376E^. Plasmid shuffling generated strains and growth behavior assessed on fermentative and respiratory carbon sources at low (23 °C) and high (37–38 °C) temperature. **c** Immunodecoration of mitochondria isolated from strains shown in **b** after cell growth for 10 h at 37 °C under respiratory conditions (non-fermentable carbon source). *i* processing intermediate, *m* mature protein, *p* precursor

MIP and its yeast homologue Oct1 are highly conserved (Additional file 1: Figure S1). Therefore, we employed a yeast model system to assess the effects of the disease mutations on MIP in vivo. We introduced the Oct1 mutations by site-directed mutagenesis and expressed the wild type and disease mutants from a plasmid under the endogenous promoter in an *OCT1* deletion strain [12]. Mitochondria were isolated and analyzed for Oct1 processing defects by SDS-PAGE and immunodecoration (Fig. 3a). Re-expression of the wild-type Oct1 protein rescued the processing defect of the Oct1 substrates Mdh1 and Sdh4 (Fig. 3a, lanes 1 and 4) while yeast cells transformed with the empty vector (e.v.) as control showed complete accumulation of Oct1 processing intermediates (Fig. 3a, lanes 2 and 5). Mutation of Oct1 at position L83Q (MIP L71Q) also fully abolished Oct1 processing (Fig. 3a, lane 3). Immunodecoration using an antibody specific to Oct1 revealed that expression of the L83Q mutant does not result in detectable Oct1 protein levels in mitochondria. Consequently, the L83Q mutant mimics an *OCT1* deletion phenotype. Mutations of L339F (MIP L306F, Fig. 3a, lane 6) and K376E (MIP K343E, Fig. 3a, lane 7) showed increased accumulation of Oct1 processing intermediates for Mdh1 and Sdh4, indicating an impaired Oct1 proteolytic function in these mutants in vivo. In contrast, non-Oct1 substrates (controls) were not changed and the protein levels of these two Oct1 mutants were comparable to those of the wild type.

Deletion of Oct1 in yeast results in loss of mitochondrial DNA. As a consequence *OCT1* deletion strains are not viable on non-fermentable carbon sources (where mitochondrial respiration is essential for cell viability). Several of the known Oct1 substrates, e.g., Rip1 and Cox4, are subunits of the respiratory chain complexes or the mitochondrial ribosome, e.g., Mrp21, and are, therefore, required for survival under respiratory growth conditions. In order to analyze the effect of the Oct1 mutants under respiratory conditions, we generated yeast cells expressing the various Oct1 mutants by plasmid shuffling (see “Methods” for details). The approach yielded viable strains for the L83Q, L339F, and K376E Oct1 mutants. (However, due to the lack of Oct1 in the mitochondria of the L83Q strain, growth under respiratory growth assessment was not possible.) While both Oct1 L339F and K376E expressing strains showed no growth defect on fermentative carbon sources, both strains displayed a severe growth defect under respiratory conditions at higher temperature (Fig. 3b). We isolated mitochondria from these strains after growth at a non-permissive temperature for 10 h and analyzed the protein steady state levels by SDS-PAGE and immunodecoration (Fig. 3c). In both mutants we found strong accumulation of the processing intermediates of Mdh1, Sdh4, Cox4, Mdj1, Prx1, and Rip1, revealing a severe decrease of Oct1 activity. The processing defect was most pronounced in the K376E mutant, in which the mature form of Rip1 was virtually absent. Control proteins, which are not substrates of Oct1, were not affected. Taken together, the analyses of the Oct1 mutations in vivo under respiratory conditions demonstrate a strong accumulation of processing intermediates of Oct1 substrates inside mitochondria, indicating a decreased proteolytic activity of Oct1.

**Fig. 4:**
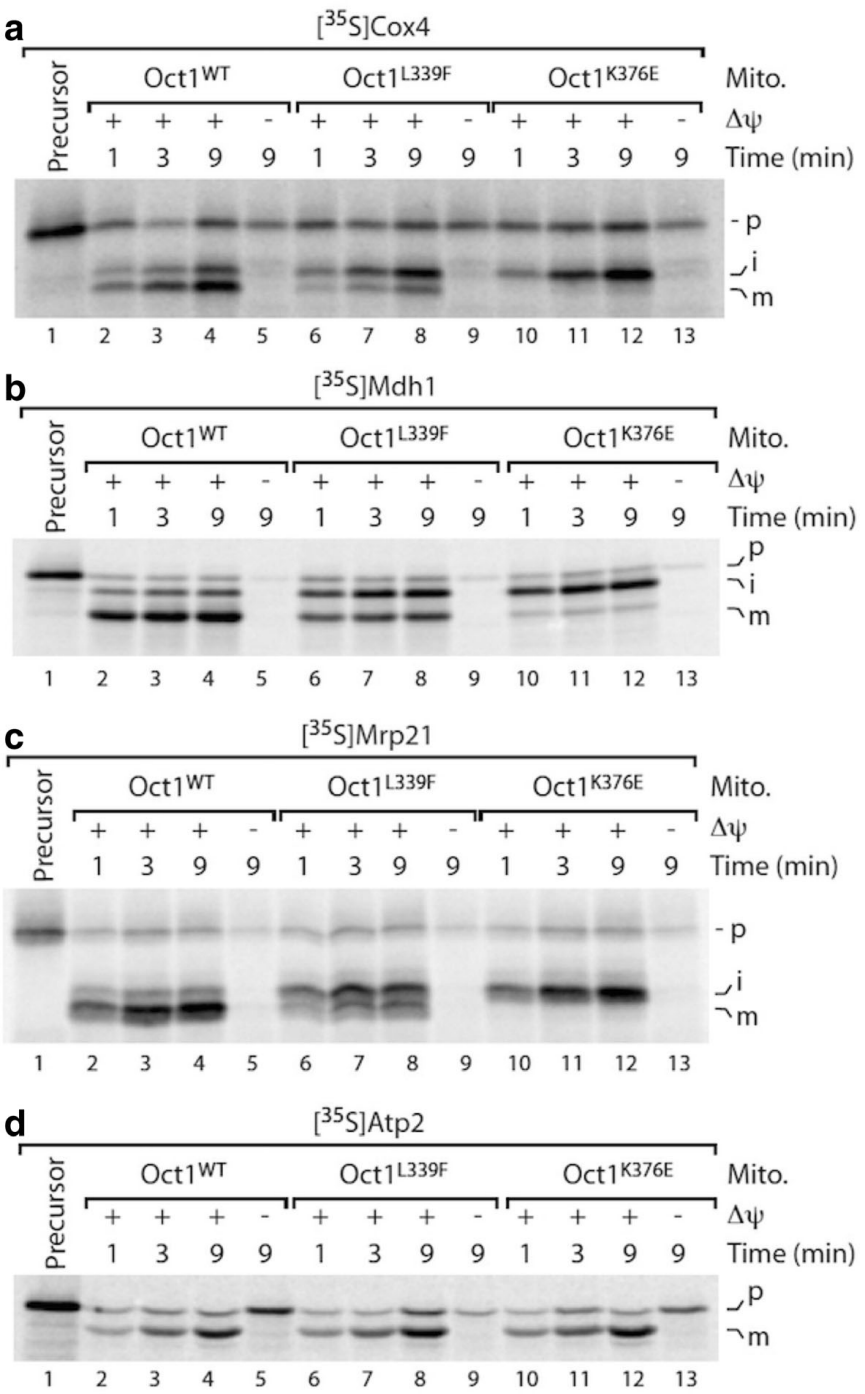
*In organello* processing activity of Oct1^L339F^ and Oct1^K376E^ mutants. **a**–**c** In vitro import and processing of radiolabeled Mdh1, Cox4, and Mrp21 preproteins in isolated mitochondria (*Mito.*) from indicated mutant strains compared to wild type (*Oct1* ^*WT*^). **d** In vitro import and processing of the Oct1-independent preprotein Atp2. The reaction was performed as in **a**–**c**. *Δψ* is the mitochondrial membrane potential. *i* processing intermediate, *m* mature protein, *p* precursor

In order to directly analyze Oct1 activity in the mutant strains in vitro, we generated [^35^S]radiolabeled precursor proteins of the Oct1 substrates Mdh1, Cox4, and Mrp21 by in vitro transcription/translation and imported them into isolated mitochondria. Samples were separated via SDS-PAGE and the different precursor processing steps were monitored by autoradiography [12, 31]. For all three precursor proteins the Oct1-mediated processing of the intermediate (i) forms to the mature (m) protein was severely impaired (Fig. 4a-c). Strikingly, the more affected Oct1 processing activity in the K376E mutant mitochondria correlated with the stronger accumulation of precursor intermediates in the mutant cells in vivo (Fig. 3c). Processing of the Atp2 precursor, which is cleaved by MPP but not Oct1, was not affected (Fig. 4d). Thus, our approach revealed that both mutations L339F and K376E diminish the proteolytic activity of Oct1 not only in vivo but also in a direct *in organello* processing assay.

## Discussion

The application of WES in clinical practice has led to the identification of novel Mendelian disorders [26, 35, 36]. We have identified four individuals with rare biallelic variants in *MIPEP* who presented with a syndrome of LVNC, DD, seizures, hypotonia, cataracts, and infantile death. Three out of four patients (75 %) have passed away in the first 3 years of life. This demonstrates the association between pathogenic biallelic variants in *MIPEP* and LVNC and early childhood death [37].

Mitochondrial presequence proteases are essential to maintain a functional mitochondrion. The majority of imported mitochondrial preproteins carry N-terminal presequences as targeting signals and require proteolytic cleavage by presequence proteases. While some mitochondrial proteins only require one step of presequence cleavage by MPP, approximately 25 % of proteins with the N-terminal targeting sequence require a second step of cleavage by MIP or XPNPEP3/Icp55. In this 25 %, the MPP processing intermediates carry destabilizing N-terminal amino acids and are subject to rapid degradation [12, 14, 15]; removal of an additional octapeptide by Oct1 or a single amino acid by XPNPEP3/Icp55 reveals stabilizing N-terminal residues following a mitochondrial *N-end rule* [12, 14, 16, 38]. The proteolytic action of Oct1 and XPNPEP3/Icp55 is required, therefore, to maintain a stable mitochondrial proteome (Fig. 1).

Functional analysis of the Oct1 L306F/L339F and K343E/K376E mutants revealed a severe decrease in Oct1 processing activity, as demonstrated by accumulation of processing intermediates. These processing intermediates included subunits of ETC complexes Sdh4 (succinate dehydrogenase, subunit of respiratory chain complex II and a novel Oct1 substrate), Rip1 (Rieske iron-sulfur-protein, subunit of respiratory chain complex III), Cox4 (cytochrome c oxidase, subunit 4 of respiratory chain complex IV), the citrate cycle enzyme Mdh1 (mitochondrial malate dehydrogenase), the ribosomal subunit Mrp21, the peroxiredoxin Prx1, and the mtHsp70 co-chaperone Mdj1 (also a novel Oct1 substrate) (Figs. 3 and 4), which showed strong accumulation in strains expressing the mutant versions of Oct1 (L339F and K376E). Since most of these proteins either directly or indirectly influence mitochondrial respiratory chain activity, it is highly likely that MIP/Oct1 defects affect mitochondrial bioenergetics. Therefore, defects in Oct1 activity might impact on respiratory chain activity.

Analysis of the protein levels of the Oct1 mutant L83Q revealed that the protein was not detectable in isolated mitochondria; the Oct1 L83Q mutant consistently mimicked an *OCT1* deletion phenotype (Fig. 3). This could be caused by decreased import rates or rapid turnover of the mutant protein upon synthesis or translocation into mitochondria.

Our functional data are analogous to the previous experiments in yeast illuminating the consequences of loss of Oct1 activity. Previously it has been demonstrated that the deletion of the yeast homolog of *MIPEP*, Oct1, disrupted iron-sulfur (FeS), Cox4, and ornithine transcarbamoylase (OTC) protein maturation. Conversely, the reintroduction of Oct1 into the yeast led to the resumption of FeS, Cox4, and OTC protein processing [39]. Furthermore, the biochemical and metabolic consequences of Oct1 deletion include a significant reduction in NADH dehydrogenase activity and succinate dehydrogenase activity that has been explained by a partial loss of mitochondrial respiratory component function [39]. Subsequent experiments in a yeast experimental model revealed other functions of Oct1, including its role in processing of mitochondrial proteins involved in regulation of mtDNA replication [39, 40]. The biochemical evaluations of Oct1 in yeast have shed light on its protein structure, revealing a zinc-binding domain involved in its cleavage activity. Mutations in the zinc-binding domain disrupt the Oct1 catalytic activity; four mutations in the zinc-binding domain, including p.H558R, p.E559D, p.H565R, and p.E587D, caused global loss of Oct1 activity in yeast [41].

Recessive mutations in *XPNPEP3* (MIM 613159) have been reported to cause nephronophthisis-like nephropathy 1 (NPHPL1) [42]. Mutations in the alpha subunit of MPP, encoded by *PMPCA* (MIM 213200), have been recently identified to cause an autosomal recessive form of non-progressive cerebellar ataxia [43]. Copy number variations and SNVs have been reported to alter the inner mitochondrial membrane peptidase subunit 2 encoded by *IMMP2L*, causing neurodevelopmental disorders and age-associated neurodegeneration, respectively [44, 45]. Our identification of pathogenic variants in *MIPEP* in patients with LVNC and neurologic abnormalities illustrates the fundamental role of presequence proteases in mitochondrial protein processing and function and their contribution to human disease.

## Conclusions

Our study reveals the first link between SNVs in the gene of the mitochondrial intermediate peptidase *MIPEP* and severe forms of LVNC. Phenotypic evaluation following the identification of biallelic *MIPEP* variants in four patients identified a shared and rare syndrome of LVNC, DD, seizures, hypotonia, cataracts, and infantile/early childhood death. Experimental data in yeast support the pathogenicity of these variants and indicates that the mechanism of action is loss of function of MIP. The identification and description of the patients’ genotype and phenotype together with functional biochemical analysis provide insights into the consequences of MIP dysfunction. Identification of this severe, early-onset condition expands the phenotypic spectrum associated with loss of mitochondrial presequence protease function to include cardiomyopathy and neurologic impairment.

## Additional files

Additional file 1: Figure S1. Sequence alignment of human and yeast MIP. (JPG 890 kb)
Additional file 2: Figure S2. Digital droplet PCR (ddPCR) was performed using the QX200™ AutoDG™ Droplet Digital™ PCR System from Bio-Rad following the manufacture’s protocols. Briefly, a 20-μL mixture was set up for each PCR reaction, containing 10 μL of 2× Q200 ddPCR EvaGreen Supermix, 0.25 μL of KpnI restriction enzyme (NEB, catalog number R0142S), 0.25 μL of each primer (10 μM) and 20 or 40 ng of genomic DNA. Reaction mixture was incubated at 37 °C for an hour for enzymatic digestion, following by automatic droplet generation, PCR reaction, and droplet reading. Cycling conditions for PCR were: 5 min at 95 °C, 40 cycles of 30 s at 95 °C/1 min at 60 °C/2 min at 72 °C, 5 min at 4 °C, 5 min at 90 °C, and finally infinite hold at 4 °C. Ramp rate was set for 2 °C per second for all steps. Data were analyzed using QuantaSoft^TM^ Software from Bio-Rad and concentrations of positive droplets (number of positive droplets per microliter of reaction) were obtained for each PCR reaction. Raw data of ddPCR and primer sequences are shown. A primer pair targeting *MIPEP* around chr13: 24436467 and two control primer pairs targeting copy number-neutral regions were used to perform ddPCR in the proband. Absolute positive droplet concentrations (copies/μL) are plotted from ddPCR results of the three primer pairs. Similar positive droplet concentrations were observed from ddPCR performed using primers targeting *MIPEP* and the two control primer pairs for both 20 ng genomic DNA input (around 40 copies/μL) and 40 ng genomic DNA input (around 80 copies/μL). This indicates that there was no copy number difference comparing *MIPEP* around chr13: 24436467 to the copy number-neutral control regions; therefore, no deletion was detected. Corresponding raw data of ddPCR and primer sequences are shown in (Figure S2). *Ctrl* control. (JPG 471 kb)
Additional file 3: Figure S3. Maternally inherited 1.4-Mb CNV deletion in patient 4 and known genes within the deleted interval. (JPG 883 kb)
Additional file 4: Table S1. Summary for clinical testing of mitochondrial electron transport chain (ETC) results in patients with *MIPEP* variants. *NA* not applicable, *SD* standard deviation. (JPG 852 kb)

## Abbreviations

ALT: Alanine transaminase
AST: Aspartate transaminase
BHCMG: Baylor Hopkins Center for Mendelian Genomics
CNV: Copy number variant
DD: Developmental delay
ETC: electron transport chain
ExAC: Exome Aggregation Consortium
IRB: Institutional review board
LVNC: Left ventricular non-compaction
MIM: Mendelian Inheritance in Man
MIP: Mitochondrial intermediate peptidase
MPP: Mitochondrial processing peptidase
MRI: Magnetic resonance imaging
SNV: Single nucleotide variant
WES: Whole exome sequencing
WT: Wild type
