# Supplementary figures and images for: MIPEP recessive variants cause a syndrome of left ventricular non-compaction, hypotonia, and infantile death
Source: Genome Med. 2016 Nov 1;8:106. doi: 10.1186/s13073-016-0360-6 (PMC5088683; doi:10.1186/s13073-016-0360-6)

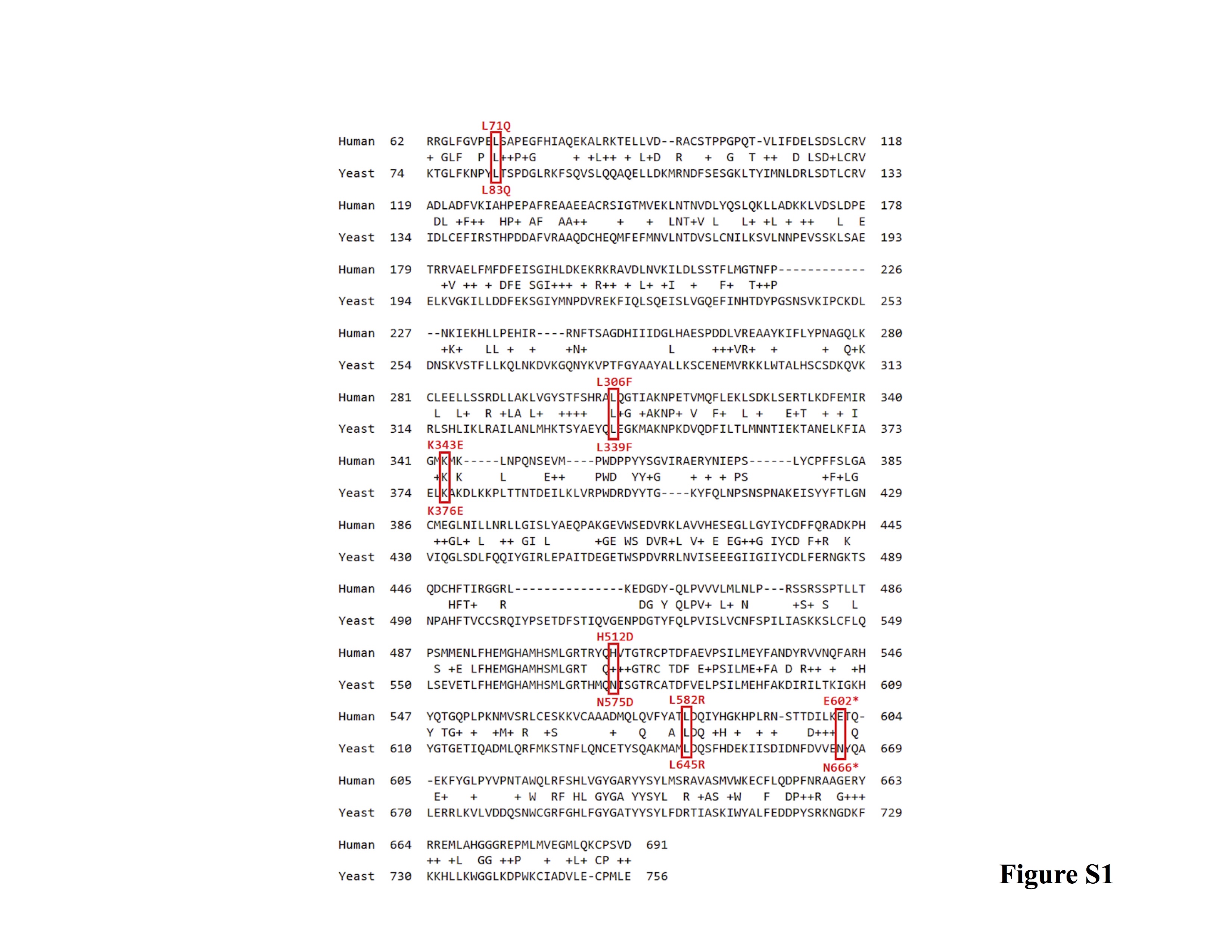

Supplement: Additional file 1: Figure S1. — Sequence alignment of human and yeast MIP. (JPG 890 kb) [file 13073_2016_360_MOESM1_ESM.jpg]

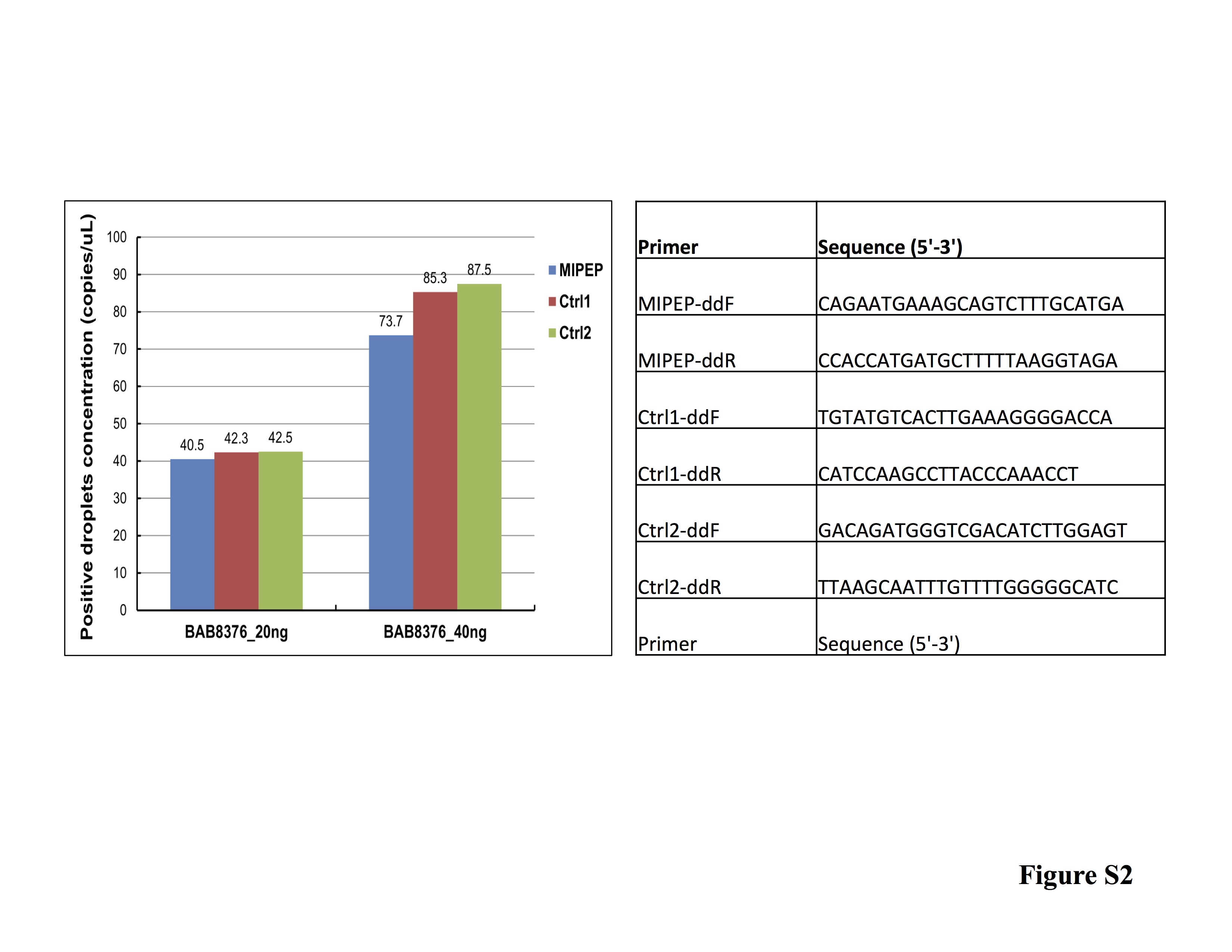

Supplement: Additional file 2: Figure S2. — Digital droplet PCR (ddPCR) was performed using the QX200™ AutoDG™ Droplet Digital™ PCR System from Bio-Rad following the manufacture’s protocols. Briefly, a 20-μL mixture was set up for each PCR reaction, containing 10 μL of 2× Q200 ddPCR EvaGreen Supermix, 0.25 μL of KpnI restriction enzyme (NEB, catalog number R0142S), 0.25 μL of each primer (10 μM) and 20 or 40 ng of genomic DNA. Reaction mixture was incubated at 37 °C for an hour for enzymatic digestion, following by automatic droplet generation, PCR reaction, and droplet reading. Cycling conditions for PCR were: 5 min at 95 °C, 40 cycles of 30 s at 95 °C/1 min at 60 °C/2 min at 72 °C, 5 min at 4 °C, 5 min at 90 °C, and finally infinite hold at 4 °C. Ramp rate was set for 2 °C per second for all steps. Data were analyzed using QuantaSoftTM Software from Bio-Rad and concentrations of positive droplets (number of positive droplets per microliter of reaction) were obtained for each PCR reaction. Raw data of ddPCR and primer sequences are shown. A primer pair targeting MIPEP around chr13: 24436467 and two control primer pairs targeting copy number-neutral regions were used to perform ddPCR in the proband. Absolute positive droplet concentrations (copies/μL) are plotted from ddPCR results of the three primer pairs. Similar positive droplet concentrations were observed from ddPCR performed using primers targeting MIPEP and the two control primer pairs for both 20 ng genomic DNA input (around 40 copies/μL) and 40 ng genomic DNA input (around 80 copies/μL). This indicates that there was no copy number difference comparing MIPEP around chr13: 24436467 to the copy number-neutral control regions; therefore, no deletion was detected. Corresponding raw data of ddPCR and primer sequences are shown in (Figure S2). Ctrl control. (JPG 471 kb) [file 13073_2016_360_MOESM2_ESM.jpg]

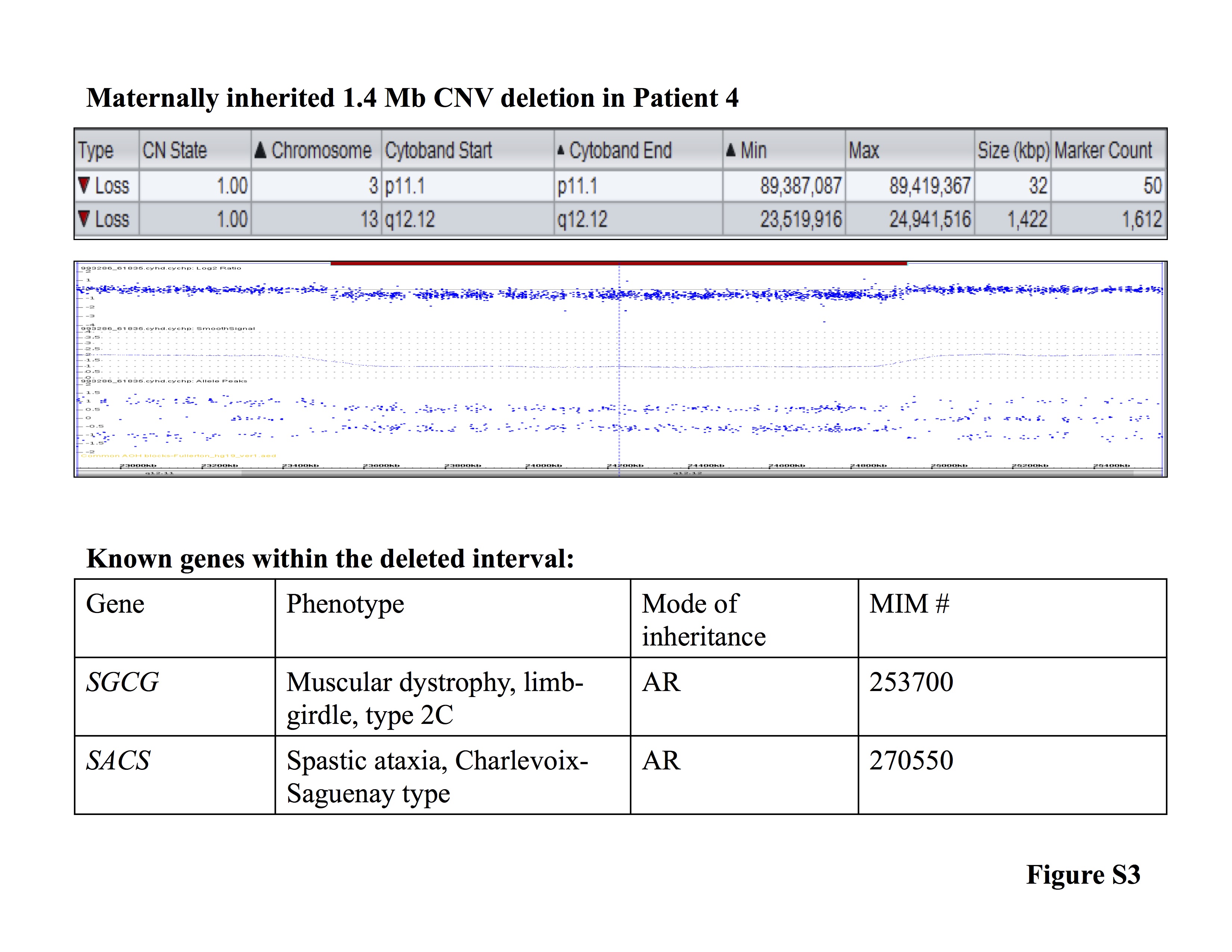

Supplement: Additional file 3: Figure S3. — Maternally inherited 1.4-Mb CNV deletion in patient 4 and known genes within the deleted interval. (JPG 883 kb) [file 13073_2016_360_MOESM3_ESM.jpg]

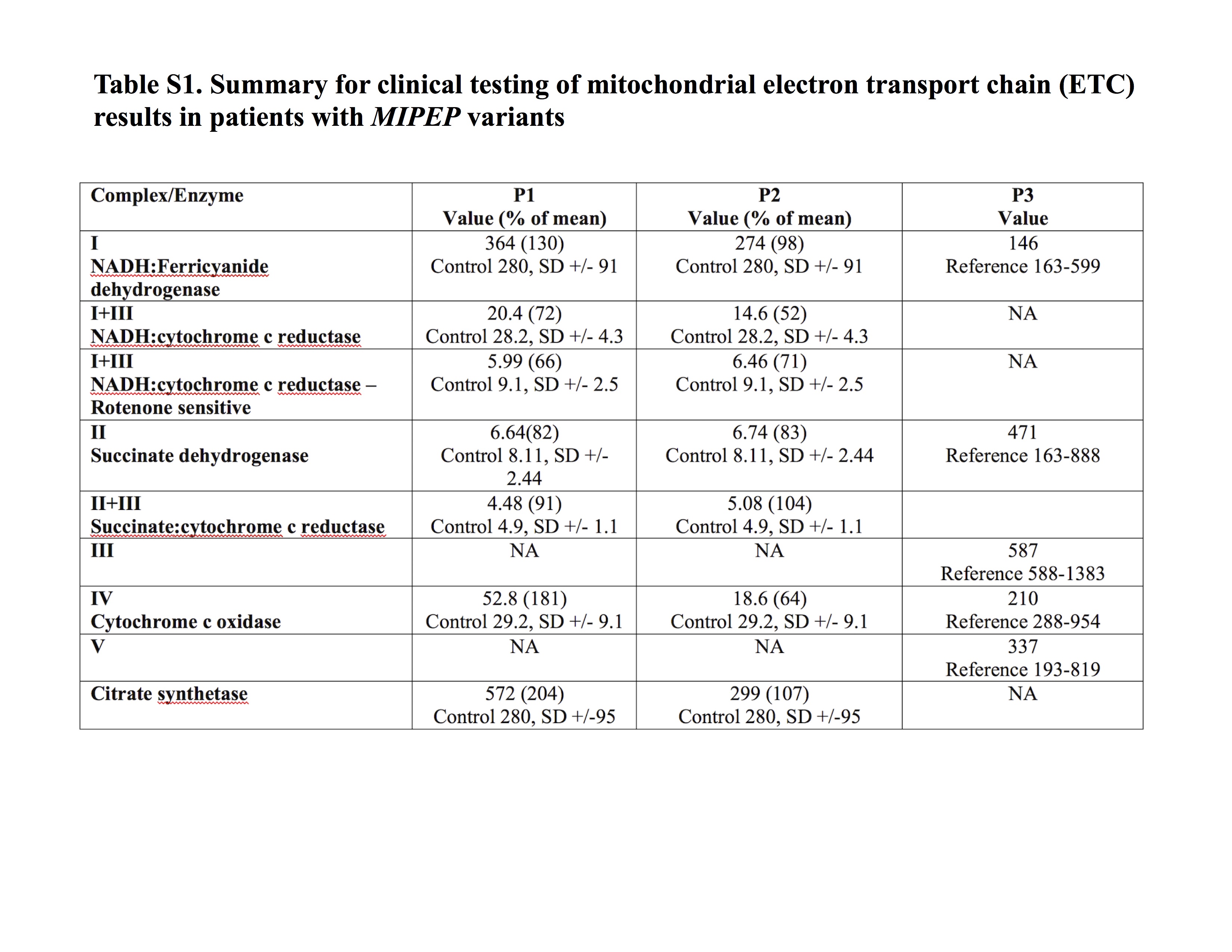

Supplement: Additional file 4: Table S1. — Summary for clinical testing of mitochondrial electron transport chain (ETC) results in patients with MIPEP variants. NA not applicable, SD standard deviation. (JPG 852 kb) [file 13073_2016_360_MOESM4_ESM.jpg]
